# The presence and co-incidence of geriatric syndromes in older patients with mild-moderate Lewy body dementia

**DOI:** 10.1186/s12883-022-02897-7

**Published:** 2022-09-19

**Authors:** Jinghuan Gan, Zhichao Chen, Shuai Liu, Zhihong Shi, Yiming Liu, Xiao-Dan Wang, Chunyan Liu, Yong Ji

**Affiliations:** 1 Department of Neurology, Beijing Tiantan Hospital, Capital Medical University, China National Clinical Research Center for Neurological Diseases, 119 Nansihuan West Road, Fengtai District, Beijing, 100070 China; 2 Department of Neurology, Beijing Friendship Hospital, Capital Medical University, Beijing, China; 3 Department of Neurology, Tianjin Huanhu Hospital, Tianjin Key Laboratory of Cerebrovascular and of Neurodegenerative Diseases, Tianjin Dementia Institute, 6 Jizhao Road, Jinnan District, Tianjin, 300350 China; 4 Department of Neurology, Qilu hospital, Shandong University, Jinan, China; 5 Department of Neurology, Aviation General Hospital, Beijing, China

**Keywords:** Dementia with Lewy bodies, Parkinson’s disease dementia, APOE ε4 carriers, Fall, Neuropsychiatric symptom

## Abstract

**Introduction:**

Geriatric symptoms are common in dementia cases, while few studies have focused on these symptoms in Lewy body dementia (LBD). The purpose of this study is to investigate the distributions of Apolipoprotein E (APOE) ε4 and geriatric symptoms, and explore their associaitons in Dementia with Lewy bodies (DLB) and Parkinson’s disease dementia (PDD).

**Methods:**

A retrospective study with 185 mild-moderate probable DLB (*n* = 93) and PDD (*n* = 92) patients was assigned. Demographic and clinical characteristics, neuropsychological assessments, and APOE genotypes were recorded. Description, correlation and logistic regression models were used to analyze the presence of geriatric symptom complaints and their associations with APOE ε4.

**Results:**

DLB patients displayed more frequency of fluctuating cognition, visual hallucination, rapid eye movement sleep behavior disorder, delusion, depression, anxiety, apathy, and loss of appetite, whereas the PDD cases had constipation, fear of falling, and insomnia more frequently. The APOE ε4 allele was more common in DLB than PDD (29.9% vs. 7.0%, *p* < 0.001), and the patients with DLB + APOE ε4 (+) were presented more delusions (*p* = 0.005) and apathy (*p* = 0.007) than patients with PDD + APOE ε4 (+). We also found that the APOE ε4 allele was significantly associated with hyperhidrosis (OR = 3.472, 95%CI: 1.082–11.144, *p* = 0.036) and depression (OR = 3.002, 95%CI: 1.079–8.353, *p* = 0.035) in DLB patients, while there were no significant associations between APOE ε4 allele and the age at visit, the age at onset, scores of MDS-UPDRS III, H&Y stage, ADL, MMSE, MOCA and NPI, as well as the presences of fluctuating cognition, VH, parkinsonism and RBD in both groups.

**Conclusion:**

The presence and co-incidence of geriatric symptoms are common in patients with mild-moderate LBD. The presence of APOE ε4 allele is associated with hyperhidrosis and depression, but not global cognition, activitives of daily life, motor function and other neuropsychitric symptoms in DLB. These findings improve the awareness of geriatric symptoms, and contribute to the healthcare management of mild-moderate DLB and PDD.

**Supplementary Information:**

The online version contains supplementary material available at 10.1186/s12883-022-02897-7.

## Introduction

Lewy body dementia (LBD) is the second most common neurodegenerative dementia, comprises both dementia with Lewy bodies (DLB) and Parkinson’s disease dementia (PDD) [1]. Though the two diseases are distinguished clinically from “1-year rule” (in PDD the motor symptoms precede the onset of dementia by at least 1 year) [2], their association remains to be clarified because of pathological and genetic overlap on Lewy body disease continuum [3].

LBD patients present with a wide range of motor and nonmotor (cognitive, neuropsychiatric, sleep, autonomic nervous dysfunction) symptoms. Numerous studies have paid attention to “geriatric syndromes” of dementia older adults, which can increase the disability and dependence [4], play an important role in dementia diagnoses and management as well. Previous studies point that polypharmacy [5], falls [6], fear of falling (FoF) [7], frailty [8], orthostatic hypotension (OH) [9], urinary and fecal incontinence [10], depression [11], and sleep distribution [2] may increase cognitive impairment, neuropsychiatric symptoms, and dependence on daily living, even in early-stage dementia subtypes [12]. What’s more, APOE ε4 allele is a strong risk factor across the Lewy body disease spectrum, and it has been confirmed to be associated with the occurrence of LBD, and can accelerate LBD progression and cause poor prognosis [13–15]. Despite these associations, it remains unknown the role of APOE ε4 on the geriatric symptoms in mild-moderate LBD patients.

Therefore, the purpose of this study is to compare the presences of APOE genotypes and geriatric symptoms, as well as explore the associations between them in mild-moderate LBD patients. The recognition and awareness of geriatric symptoms will markedly improve the early identification and accurate treatment of DLB and PDD, which may contribute to the healthcare management.

## Material and methods

### Participants

A total of 3221 subjects were seen by a cognitive impairment specialty clinical service in the Tianjin Huanhu Hospital, Tianjin, China from June 2017 to December 2020.

The panel made their diagnosis based on the corresponding diagnostic criteria, namely DLB was based on 2017 criteria by McKeith et al. [2], PDD was diagnosed according to the criteria proposed by the Movement Disorder Society in 2007 [16]. A probable DLB diagnosis can be made with two or more core symptoms together with or without indicative biomarkers, or only one core symptom together with one or more indicative biomarkers. PDD patients were diagnosed according to the clinical criteria for probable PDD [16]. International consensus suggests that DLB should be diagnosed when cognitive impairment precedes parkinsonism or begins within a year of parkinsonism and PDD should be diagnosed when parkinsonism precedes cognitive impairment by more than 1 year. Blood tests, the APOE genotypes, neuroimaging (including computerized tomography scans and/or magnetic resonance imaging), and positron emission computed tomography were performed, if necessary, to make the diagnosis [17]. Therefore, DLB and PDD mentioned in the study were from a probable diagnosis. All included participants with DLB and PDD had the score of Clinical Dementia Rating (CDR) between 1 and 2 (CDR = 1.0, CDR = 2.0), and were at least 50 years old.

**Fig. 1:**
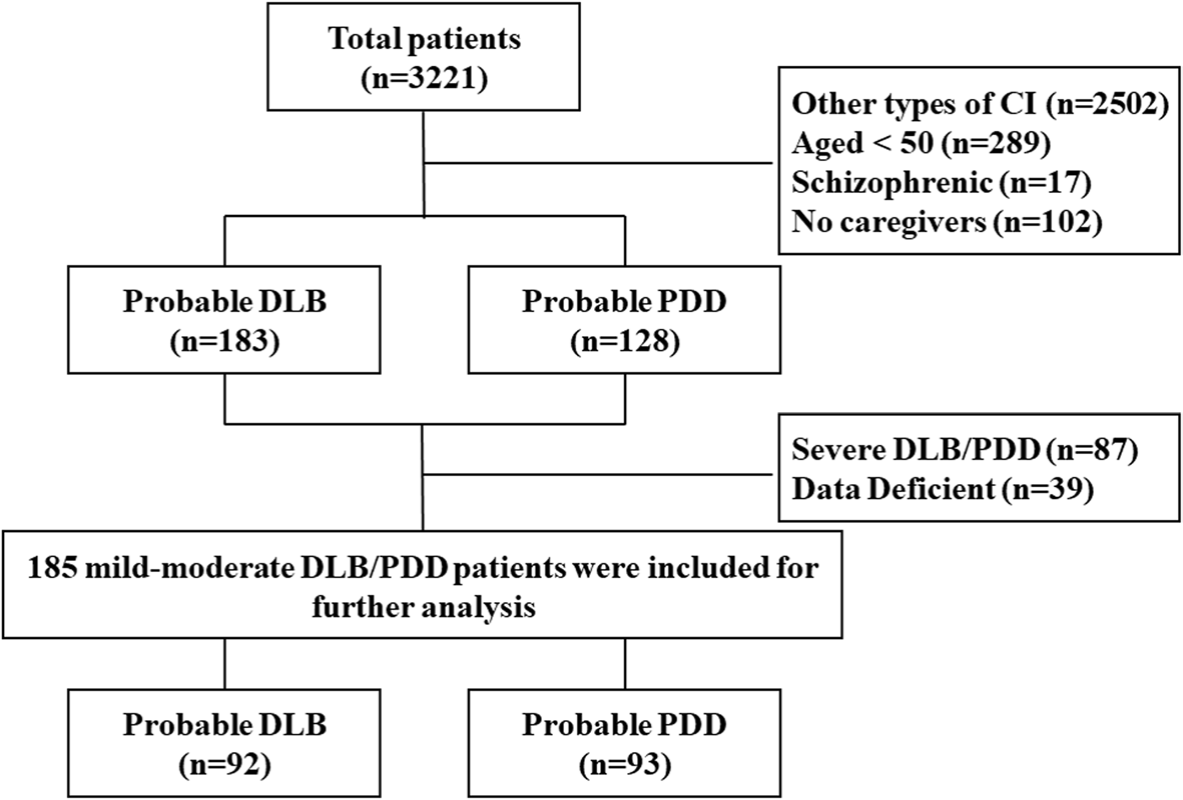
Flow chart of this study. Abbreviations: CI, cognitive impairment; DLB, dementia with Lewy bodies; PDD, Parkinson’s disease dementia

Diagnoses were confirmed by a two-specialist panel according to the basic diagnostic flowchart (the same as our previous study [17]), if there was disagreement between them, the subject would be excluded. Participants were also excluded if they had a major concurrent psychiatric illness or a history of other serious neurological illness, including stroke or alcohol/substance abuse history. Because the Neuropsychiatric Inventory (NPI) should be conducted with the caregivers, patients without caregivers were also excluded. Finally, 185 mild-moderate probable LBD patients, containing 93 DLB and 92 PDD, were invited in this research (Fig. 1).

### Assessments for core clinical features

#### Fluctuating cognition

The Mayo Fluctuations Composite Scale was used to confirm the presence of cognitive fluctuations, with three or more “yes” responses required for structured questions from caregivers [18].

#### Visual hallucinations (VH)

Specifically formed and detailed VH and illusions, that were complained about by the patient and/or caregiver were determined by confirmation and quantification according to the hallucinations item of the NPI [19]. The chief complaint of seeing people, children or animals that were not present was recorded as “visual hallucination”.

#### Parkinsonism

One or more spontaneous cardinal features of parkinsonism included bradykinesia (defined as slowness of movement and decrement in amplitude or speed), rest tremor or rigidity, diagnosed by the motor section (Part III) of the Movement Disorders Society Unified Parkinson’s Disease Rating Scale (MDS-UPDRS) [20].

Rapid eye movement sleep behavior disorder (RBD): This was confirmed by caregivers who mentioned five or more behaviors that are mentioned in the RBD screening questionnaire (RBD-SQ) [21] or someone who was diagnosed using an overnight video polysomnography [22]. Totally, 37 patients with DLB and 45 patients with PDD underwent polysomnography; and 83 patients with DLB and 81 patients with PDD underwent RBD-SQ to confirm RBD.

### Assessments for geriatric symptom complaints

Demographic characteristics (age, gender, and years of education), cardiometabolic conditions [heart disease, hypertension, diabetes mellitus (DM), stroke], the history of smoking/alcohol consumption, drug prescriptions for DLB/PDD were recorded. A battery of standardized instruments, including the Chinese version of the MMSE (C-MMSE), Montreal Cognitive Assessment (MoCA), activities of daily living (ADL) and NPI, was used to evaluate cognitive function status, activities of daily living and neuropsychiatric symptoms (NPS) respectively. The scores of C-MMSE [23, 24] and MoCA [25] range 0 (severe impairment) to 30 (no impairment). The NPI [19] comprises 12 items and provided by their caregivers. Each subscale ranges between 0 (no NPS) and 12 and the total composite score between 0 (no NPS) and 144. The Chinese version of ADL (revised by Zhang et.al) [26] consists of 20 items to evaluate the function of physical self-maintenance [include eating, bathing, dressing, grooming (and shear toe nail), transferring (e.g. walk in a flat room, moving from chair to bed and return, walk up and down stairs), and using the toilet.] and instrumental activities [including use of the telephone, shopping, preparing a hot meal, taking water for cooking and bathing, doing housework, taking medication, managing financial matters, getting to places beyond walking distances, and doing laundry], with the total scores between 20 (no impairment) and 80 (severe dysfunction).

Patients were routinely evaluated the geriatric symptoms and conditions in our clinic, including OH, constipation, urinary symptoms, hyperhidrosis (HH), falls, FoF, delusion, depression, anxiety, apathy, loss of appetite, insomnia, and excessive daytime sleepiness (EDS). The measurement instruments were described as below:

- OH: Over 20 mmHg decrease in systolic blood pressure or > 10 mmHg decrease in diastolic blood pressure within 3 min of standing or have symptoms of postural dizziness.
- Constipation: Defecation less than three times a week, hard stool, having to strain to pass stools.
- Urinary symptoms: Complaining of frequent or urgent micturition, dysuria, or urinary incontinence, except for urinary tract infection.
- HH: Suffering from excessive sweating required to regulate normal body temperature during the day or at night.
- Falls: Having fallen in the previous year except for tripping on a rug and slipping on wet floor.
- Fear of falling: Fearing of falling in daily activities and having The Falls Efficacy Scale International > 16.
- Delusion: The caregivers responded “Yes” to items 1 on the NPI.
- Depression: The caregivers responded “Yes” to items 4 on the NPI.
- Anxiety: The caregivers responded “Yes” to items 5 on the NPI.
- Apathy: The caregivers responded “Yes” to items 7 on the NPI.
- Loss of appetite: The caregivers reported that the patient had a loss of appetite and a lack of desire to eat, then responded “Yes” to items 12–1 on the NPI.
- Insomnia: Insomnia Severity Index score ≥ 8.
- Excessive daytime sleepiness: Epworth Sleepiness Scale score ≥ 11.

### APOE genotyping

We used peripheral blood to extract genomic DNA, and polymerase chain reaction was amplified to determine the APOE gene in this study. All the detailed method had been described at our previous study [27]. We were sure about all genotypes without knowledge of the patient status.

### Statistical analyses

Descriptive analyses were conducted using frequency for qualitative variables and mean [± standard deviation (SD)] or median [interquartile range (IQR)] for quantitative variables. Student’s t-test was run to compare the two independent groups (DLB and PDD) for normally distributed data and a Mann-Whitney U test was used for nonparametric data. The comparisons among the four groups [DLB with APOE ε4 (+), DLB with APOE ε4 (−), PDD with APOE ε4 (+), PDD with APOE ε4 (−)] were conducted by Mann–Whitney U tests and Bonferroni corrections were applied. Qualitative variables were assessed using a chi-squared test. Analysis of correlation was made by using Pearson and Partial (adjusted gender, education, habits of smoking and alcohol consumption, heart disease, hypertension, diabetes mellitus, stroke and CDR) correlations to evaluate the correlation between APOE ε4 allele, basic and clinical characteristics in probable DLB and PDD. Logistic regression models (adjusted gender, education, habits of smoking and alcohol consumption, heart disease, hypertension, diabetes mellitus, stroke and CDR) were used to analyze the associations between APOE ε4 allele and geriatric symptoms, and the results were recorded with odds ratios (ORs) and 95% confidential interval (95% CI).

For the statistical analyses, the IBM Statistical Package for the Social Sciences (SPSS) for Windows (version 22.0; IBM Corporation, Armonk, NY) was used. Adjusted odds ratios (OR) are presented with 95% confidence interval (95% CI). A *p* - value of less than 0.05 was considered significant. All tests were performed bilaterally.

## Results

### Clinical characteristics

A total of 185 patients, 92 with mild-moderate DLB and 93 with mild-moderate PDD, were included in the study (Table 1). The median and mean ages of DLB patients, both age at visit and onset, were older, and DLB patients had higher proportions of hypertension (32.6% vs. 17.2%, *p* = 0.015) and DM (23.9% vs. 10.8%, *p* = 0.018), as well as were more APOE ε4 carriers (57.6% vs. 12.9%, *p* < 0.001) than PDD patients. Comparing to patients with PDD, the DLB cases displayed more frequency of fluctuating cognition (28.3% vs. 9.7%, *p* = 0.001), VH (47.8% vs. 14.0%, *p* < 0.001) and RBD (55.4% vs. 22.6%, *p* < 0.001) at the time of diagnosis, while there was no significant difference in APOE ɛ4 allele subgroups (Additional file, Table S1).

**Table 1:** Sample characteristics

	DLB (*n* = 92)	PDD (*n* = 93)	*x*^2^/Z-score	*P*-value
Gender, n (%)			0.046	0.830
Male	50 (54.3%)	52 (55.9%)		
Female	42 (45.7%)	41 (44.1%)		
Age at visit, years, median (IQR)	71 (66,78)	67 (62,74)	−3.900	**0.000**
Age at onset, years, median (IQR)	69 (63,75)	63 (59,71)	−3.456	**0.001**
Education, years, median (IQR)	9 (9,15)	9 (6,12)	−2.142	**0.032**
Marital status, n (%)			0.144	0.705
Married	67 (72.8%)	70 (75.3%)		
Widowed	20 (21.7%)	23 (24.7%)		
Divorced	1 (1.1%)	0 (0.0%)		
Single	4 (4.3%)	0 (0.0%)		
Smoking, yes, n (%)	25 (27.2%)	20 (21.5%)	0.807	0.369
Alcohol consumption, yes, n (%)	20 (21.7%)	15 (16.1%)	0.949	0.330
Cardiometabolic conditions, n (%)				
Heart disease	21 (22.8%)	31 (33.3%)	2.527	0.112
Hypertension	30 (32.6%)	16 (17.2%)	5.874	**0.015**
DM	22 (23.9%)	10 (10.8%)	5.599	**0.018**
Stroke	12 (13.0%)	8 (8.6%)	0.946	0.331
APOE genotype frequency, n (%)				
2/2	1 (1.1%)	2 (2.2%)	0.328	1.000
2/3	3 (3.3%)	8 (8.6%)	2.359	0.212
2/4	0 (0.0%)	1 (1.1%)	0.995	1.000
3/3	35 (38. 0%)	71 (76.3%)	27.728	**0.000**
3/4	51 (55.4%)	10 (10.8%)	41.779	**0.000**
4/4	2 (2.2%)	1 (1.1%)	0.350	0.621
APOE allele frequency, n (%)				
ε2	5 (2.7%)	13 (7.0%)	3.647	0.056
ε3	124 (67. 4%)	160 (86.0%)	17.995	**0.000**
ε4	55 (29.9%)	13 (7.0%)	32.342	**0.000**
APOE ε4 carriers, n (%)			40.557	**0.000**
Yes	53 (57.6%)	12 (12.9%)		
No	39 (42.4%)	81 (87.1%)		
Drug prescriptions, n (%)				
ChEIs	63 (68.5%)	9 (9.7%)	67.266	**0.000**
Anti-Parkinsonism	11 (12.0%)	50 (53.8%)	36.575	**0.000**
Antipsychotics	18 (19.6%)	3 (3.2%)	12.270	**0.000**
Core features, n (%)				
FLC	26 (28.3%)	9 (9.7%)	10.412	**0.001**
VH	44 (47.8%)	13 (14.0%)	24.855	**0.000**
Parkinsonism	44 (47.8%)	93 (100.0%)	54.914	**0.000**
RBD	51 (55.4%)	21 (22.6%)	21.000	**0.000**

*Abbreviations*: *DLB* Dementia with Lewy bodies, *PDD* Parkinson’s disease dementia, *IQR* Interquartile range, *DM* Diabetes mellitus, *APOE* Apolipoprotein E, *ChEIs* Cholinesterase inhibitors, *FLC* Fluctuating cognition, *VH* Visual hallucination, *RBD* Rapid eye movement sleep behavior disorder

The global and sub-item scores of MMSE and MoCA were significantly higher in the PDD cases than DLB cases at their first visit (Table 2). And patients with PDD had significantly greater H&Y stage (mean score: 2.12 ± 0.64 vs. 0.98 ± 1.50, *p* = 0.000) higher UPDRS part III scores (mean score: 30.76 ± 14.48 vs. 7.80 ± 1.50, *p* = 0.000). While the patients with DLB had higher scores of NPI (mean score: 15.14 ± 13.77 vs. 2.65 ± 4.49, *p* = 0.000) and ADL (mean score: 32.77 ± 12.74 vs. 26.04 ± 10.15, *p* = 0.000). We also found that the presence of APOE ε4 allele was not significantly correlated with the age at visit, the age at onset, scores of MDS-UPDRS III, H&Y stage, ADL, MMSE, MOCA and NPI, as well as the presences of fluctuating cognition, VH, parkinsonism and RBD in both groups, except for the scores of MoCA-language (r = − 0.264, *p* = 0.016) in DLB cases and MMSE-registration (r = − 0.216, *p* = 0.038) in PDD cases (Additional file, Table S2). Besides, there were no significant associations between clinical core features of LBD and APOE ε4 allele after adjusting confounders (Additional file, Table S3).

**Table 2:** Cognitive and motor function in patients with probable DLB and PDD

Characteristics	DLB		PDD		Z-score	***P***-value
	Median (IQR)	Mean (SD)	Median (IQR)	Mean (SD)		
**MMSE**	18.00 (14.00, 24.00)	18.57 ± 5.41	24.00 (20.00, 26.00)	23.02 ± 3.51	−5.500	**0.000**
Orientation	6.00 (4.00, 9.00)	6.38 ± 2.60	9.00 (8.00, 10.00)	8.67 ± 1.48	−6.147	**0.000**
Registration	3.00 (3.00, 3.00)	2.75 ± 0.55	3.00 (3.00, 3.00)	2.88 ± 0.39	−2.053	**0.040**
Attention and calculation	2.00 (1.00, 3.00)	2.15 ± 1.57	2.00 (2.00, 4.00)	2.83 ± 1.36	−3.231	**0.001**
Recall	1.00 (0.00, 2.00)	0.95 ± 1.03	2.00 (1.00, 2.00)	1.61 ± 0.97	−4.361	**0.000**
Language	6.00 (5.00, 7.00)	5.89 ± 1.64	7.00 (5.50, 7.50)	6.48 ± 1.26	−2.326	**0.020**
Praxis	0.00 (0.00, 1,00)	0.45 ± 0.50	1.00 (1.00, 1.00)	0.54 ± 0.50	−1.248	0.212
**MoCA**	12.00 (8.00, 18.75)	13.36 ± 6.17	17.00 (13.00, 20.00)	16.37 ± 4.44	−3.727	**0.000**
Visuospatial/executive abilities	1.50 (1.00, 3.00)	1.84 ± 1.30	2.00 (1.00, 3.00)	2.13 ± 1.31	−1.646	0.100
Naming	2.00 (2.00, 3.00)	2.18 ± 0.78	2.00 (2.00, 3.00)	2.27 ± 0.85	−1.029	0.304
Attention	4.00 (2.00, 6.00)	3.92 ± 2.19	5.00 (4.00, 5.00)	4.42 ± 1.42	−1.859	0.063
Language	1.00 (0.00, 1.00)	0.91 ± 0.93	1.00 (1.00, 2.00)	1.30 ± 0.78	−3.393	**0.001**
Abstraction	0.00 (0.00, 1.00)	0.45 ± 0.67	0.00 (0.00, 1.00)	0.46 ± 0.72	−0.031	0.975
Recall	0.00 (0.00, 0.00)	0.50 ± 1.11	0.00 (0.00, 1.50)	0.80 ± 1.04	−3.138	**0.002**
Orientation	4.00 (2.00, 5.00)	3.54 ± 1.78	5.00 (4.00, 6.00)	4.99 ± 1.15	−5.655	**0.000**
**NPI**	11.00 (5.25, 22.50)	15.14 ± 13.77	1.00 (0.00, 3.50)	2.65 ± 4.49	−8.282	**0.000**
**ADL**	28.50 (23.00, 37.75)	32.77 ± 12.74	22.00 (21.00, 25.00)	26.04 ± 10.15	−5.168	**0.000**
**CDR**	1.00 (1.00, 2.00)	1.36 ± 0.48	1.00 (1.00, 1.00)	1.13 ± 0.34	−3.630	**0.000**
**MDS-UPDRS III**	3 (0.00, 12.75)	7.80 ± 1.50	27.00 (19.50, 39.00)	30.76 ± 14.48	−9.539	**0.000**
**H&Y stage**	0.00 (0.00, 2.00)	0.98 ± 1.50	2.00 (2.00, 2.00)	2.12 ± 0.64	−7.223	**0.000**

*Abbreviations*: *DLB* Dementia with Lewy bodies, *PDD* Parkinson’s disease dementia, *IQR* Interquartile range, *SD* Standard deviation, *MMSE* Mini-Mental State Examination, *MoCA* the Montreal Cognitive Assessment, *NPI* Neuropsychiatric Inventory, *ADL* Activities of daily living, *CDR* Clinical Dementia Rating, *MDS-UPDRS III* Movement Disorder Society Unified Parkinson’s Disease Rating Scale part III, *H&Y stage* Hoehn-Yahr stage

### Associations between APOE ε4 and geriatric symptom complaints in DLB and PDD

In patients with DLB, the most frequent geriatric symptom complaints were anxiety (50.0%), followed by depression (48.9%), apathy (45.7%), loss of appetite (38.0%) and delusion (34.8%), while constipation (53.8%), insomnia (49.5%), FoF (45.2%), OH (25.8%) and falls (25.8%) troubled patients with PDD mostly, as summarized in Table 3. The presence of constipation, FoF, and insomnia were more common in the PDD than DLB (*p* < 0.05), and delusion, depression, anxiety, apathy, and loss of appetite were more common in the DLB than PDD (*p* < 0.001). And the patients with DLB + APOE ε4 (+) were presented more delusions (*p* = 0.005) and apathy (*p* = 0.007) than patients with PDD + APOE ε4 (+). But there were no significant differences of geriatric symptoms between APOE ε4 carriers and APOE ε4 non-carriers in both DLB and PDD groups.

**Table 3:** Comparation of geriatric symptom complaints between probable DLB and PDD

Geriatric symptom complaints	DLB			PDD			P1	P2	P3	P4
	All (***n*** = 92)	APOE ε4 (+) (***n*** = 53)	APOE ε4 (−) (***n*** = 39)	All (***n*** = 93)	APOE ε4 (+) (***n*** = 12)	APOE ε4 (−) (***n*** = 81)				
OH	20 (21.7)	13 (24.5)	7 (17.9)	24 (25.8)	4 (33.3)	20 (24.7)	0.516	0.450	0.523	0.531
Constipation	21 (22.8)	12 (22.6)	9 (23.1)	50 (53.8)	7 (58.3)	43 (53.1)	**0.000**	0.961	0.734	0.014
Urinary Symptoms	18 (18.9)	12 (22.6)	6 (15.4)	23 (24.7)	2 (16.7)	21 (25.9)	0.398	0.386	0.488	0.649
HH	23 (25.0)	17 (32.1)	5 (12.8)	16 (17.2)	1 (8.3)	15 (18.5)	0.194	0.032	0.383	0.097
Falls	16 (17.4)	10 (18.9)	6 (15.4)	24 (25.8)	4 (33.3)	20 (24.7)	0.164	0.663	0.523	0.271
FoF	18 (19.6)	10 (18.9)	8 (20.5)	42 (45.2)	5 (41.7)	37 (45.7)	**0.000**	0.844	0.794	0.091
Delusion	32 (34.8)	23 (43.4)	9 (23.1)	5 (5.4)	0 (0.0)	5 (6.2)	**0.000**	0.043	0.376	**0.005**
Depression	45 (48.9)	30 (56.6)	15 (38.5)	22 (23.7)	4 (33.3)	18 (22.2)	**0.000**	0.085	0.398	0.145
Anxiety	46 (50.0)	28 (52.8)	18 (46.2)	17 (18.3)	3 (25.0)	14 (17.3)	**0.000**	0.527	0.519	0.081
Apathy	42 (45.7)	27 (50.9)	15 (38.5)	15 (16.1)	1 (8.3)	14 (17.3)	**0.000**	0.235	0.431	**0.007**
Loss of appetite	35 (38.0)	21 (39.6)	14 (35.9)	2 (2.2)	1 (8.3)	1 (1.2)	**0.000**	0.716	0.114	0.039
Insomnia	25 (27.2)	17 (32.1)	8 (20.5)	46 (49.5)	7 (58.3)	39 (48.1)	**0.002**	0.218	0.510	0.089
EDS	17 (18.5)	8 (15.1)	9 (23.1)	11 (11.8)	2 (16.7)	9 (11.1)	0.207	0.330	0.578	0.892

P1 means all DLBs vs. all PDDs

The comparations among the four groups--DLB with APOE ε4 (+), DLB with APOE ε4 (−), PDD with APOE ε4 (+), PDD with APOE ε4 (−) were recorded as P2, P3, P4. The P2 means DLB with APOE ε4 (+) vs. DLB with APOE ε4 (−); P3 means PDD with APOE ε4 (+) vs. PDD with APOE ε4 (−); P4 means DLB with APOE ε4 (+) vs. PDD with APOE ε4 (+), and the *P*-values were corrected by Bonferroni correction (α = 0.05/4 = 0.0125)

*Abbreviations*: *DLB* Dementia with Lewy bodies, *PDD* Parkinson’s disease dementia, *APOE* Apolipoprotein E, *OH* Orthostatic hypotension, *HH* Hyperhidrosis, *FoF* Fear of falling, *EDS* Excessive daytime sleepiness

Logistic regression models in Table 4 showed that patients APOE ε4 allele was associated with the presences of HH (OR = 3.472, 95%CI: 1.082–11.144, *p* = 0.036) and depression (OR = 3.002, 95%CI: 1.079–8.353, *p* = 0.035) in the mild – moderate DLB patients after adjusted gender, age at visit, education, habits of smoking and alcohol consumption, heart disease, hypertension, diabetes mellitus, and stroke, while there was no associations between APOE ε4 allele and geriatric symptoms in mild-moderate PDD patients.

**Table 4:** Associations between APOE ε4 allele and geriatric symptom complaints in DLB and PDD

Characteristics	DLB (APOE ε4 carriers vs. non-carriers)			PDD (APOE ε4 carriers vs. non-carriers)		
	OR	95%CI	***P***-value	OR	95%CI	***P***-value
**OH**	1.488	0.496–4.461	0.478	1.790	0.426–7.520	0.525
**Constipation**	0.977	0.341–2.800	0.961	1.435	0.383–5.383	0.592
**Urinary Symptoms**	1.452	0.448–4.702	0.534	0.773	0.141–2.244	0.767
**HH**	3.472	1.082–11.144	**0.036**	0.470	0.050–4.450	0.510
**Falls**	1.342	0.407–4.428	0.629	1.790	0.426–7.520	0.427
**FoF**	0.755	0.229–2.484	0.644	1.033	0.262–4.067	0.963
**Delusion**	1.923	0.669–5.527	0.225	0.000	0.000–0.000	0.998
**Depression**	3.002	1.079–8.353	**0.035**	2.110	0.473–9.410	0.328
**Anxiety**	1.358	0.565–3.266	0.494	1.475	0.309–7.052	0.626
**Apathy**	1.925	0.760–4.879	0.167	0.655	0.068–6.277	0.714
**Loss of appetite**	1.311	0.499–3.446	0.582	0.000	0.000–0.000	0.997
**Insomnia**	1.978	0.671–5.824	0.216	1.838	0.455–7.427	0.393
**EDS**	0.552	0.162–1.884	0.343	1.375	0.208–9.091	0.741

The models for APOE ε4 carriers vs. non-carriers in DLB and PDD groups were adjusted gender, age at visit, education, habits of smoking and alcohol consumption, heart disease, hypertension, diabetes mellitus, stroke and CDR

*Abbreviations*: *DLB* Dementia with Lewy bodies, *PDD* Parkinson’s disease dementia, *ORs* Odds ratios, *95%CI* 95% confidence interval, *APOE* Apolipoprotein E, *OH* Orthostatic hypotension, *HH* Hyperhidrosis, *FoF* Fear of falling, *EDS* Excessive daytime sleepiness, *CDR* Clinical Dementia Rating

## Discussion

Our study reveals that the geriatric symptoms, particular constipation, FoF, delusion, depression, anxiety, apathy, insomnia, loss of appetite, are highly prevalent in mild-moderate LBD patients. The APOE ε4 allele was highly significant over represented and associated with HH and depression in DLB patients. While APOE ε4 allele was not associated with global cognitive impairment, motor function, activities of daily life, as well as four clinical core features in mild-moderate LBD patients.

Despite APOE ε4 was reported to be risk factors for LBD (including DLB and PDD) in previous literature [28], the frequency of APOE ε4 carriers in DLB and PDD was not sure. The APOE ε4 carriers was about 30 - 60% in DLB [29, 30] and about 30–40% in PDD [31, 32], but there was not significantly difference between them [32]. Our data showed the frequency of APOE ε4 carriers in DLB was line with previous studies, while higher than in PDD.

In current study, we found that 28.3% DLB patients had fluctuating cognition, 47.8% had VH, and 55.4% experienced RBD, which were less prevalent than our previous research [33]. We analyzed the percentages of the core features among 37 probable DLB patients and reported that 64.9% displayed fluctuation, and 73.0% showed VH, the difference was possibly due to the mild-moderate stage of these samples. While a longitudinal prospective cohort study with 100 patients with DLB, showed that about 39% patients had VH, 46% manifested fluctuating cognition, and 77% patients experienced RBD in early stage [34]. Moreover, we didn’t find significant associations between APOE ε4 allele and core clinical features in LBD patients. APOE ε4 allele is reported to be associated with hallucinations and Alzheimer’s disease (AD)-pathological protein in parkinsonian syndromes [35, 36], patients with DLB + APOE ɛ4 showed more pronounced hallucinations in atypical Parkinson’s disease (PD) cohort [37]. Consistent with our research, Gan-Or et al. [38] reported a lack of association between the APOE ε4 allele and risk of RBD in LBD patients, and this finding was confirmed by Sunwoo et al. [39], who demonstrated no difference of APOE genotypes in patients with RBD as well as general population. And APOE ε4 allele was not associated with the incidental parkinsonism symptoms in parkinsonian syndromes, it could only derive progressive cognitive impairment but not motor progression in parkinsonian syndromes [40, 41].

We also found higher proportions of constipation, FoF, and loss of appetite in PDD, while the insomnia and NPS, such as hallucinations, delusions, anxiety, depression and apathy, were more frequent in DLB patients. Constipation is the most prominent gastrointestinal manifestation in patients with PDD and DLB, and it may cause by neuronal loss in the myenteric plexus [42]. The enteric nervous system (ENS) origin suggested that the increased intestinal permeability in PD triggered alpha-synuclein aggregation within the ENS [43], so as to affect the gastrointestinal emptying function and ultimately produced PD [44]. Gastrointestinal dysautonomia also played a possible role in the etiology of constipation in PD, which had been proved in dopamine transporter single-photon emission computed tomography imaging in Hinkle‘s research [45]. Constipation increased the risk of developing PD [46] and noting that individuals experiencing the constipation may suffered from motor symptoms of PD with a mean of 18.7 years [47]. At the same time, DLB and PDD patients also have a certain proportion of gastroparesis [48]. Gastroparesis presented as nausea, reduced appetite, early satiety, bloating, vomiting, and weight loss [49], about 25–45% PD patients reported subjective symptoms [50]. Loss of appetite may be caused by prolonged gastric emptiness, which has an adverse interaction with constipation. The NPS in mild - moderate DLB are more frequent than PDD, which is consistent with many studies [51]. Depression is particularly common in DLB and established as a supportive feature in the revised criteria for the clinical diagnoses [2], and the high depression rates assessed by NPI are also reported in several cohort patients with mild dementia [37, 52]. A cohort with 223 dementia cases [52] (56 with DLB and 11 with PDD) reported significantly more depression in DLB but there was no relationship between the presence of APOE ε4 allele and depression. However, our study revealed opposite results. The APOE ɛ4 allele was associated with depression in mild-moderate DLB after adjusted several confounders. Several studies had reported an association between APOE ε4 and depressive phenomenology [53, 54]. APOE ε4 allele can disturbed the clearness of amyloid plaques stored in limbic regions to regulate emotional processes, and the deficits if cholinergic [55] and noradrenergic [56] activities in APOE ε4 carriers might explain the more severe depressive symptoms in mild DLB.

In addition, there are few studies comparing the falls and FoF of DLB and PDD. In a prospective study involving 30 DLB and 40 PDD patients, the incidence of falls (86.8%) was higher in PDD than in DLB (69.2%) [6]. Patients with DLB sustained 6 times, and PDD sustained 20 times more falls than normal cognition older adults [57]. The old age, sleepiness, dementia, autonomic nervous dysfunction, and freezing of gait were all risk factors of falls [58]. However, the research on FoF is mostly limited to DLB and AD. In a comparative research among old adults with DLB, AD, and non-dementias, DLB patients had much higher proportion of FoF (86.9%) than those with AD (36.0%) and without dementia (37.4%) [59]. Parkinsonism, visual dysfunction (including visual hallucination, color vision impairment, visuospatial disorder, and decreased occipital lobe activity), OH, and attention disorders may provide plausible pathways causing development of FoF [12]. Patients with PDD had more proportion of insomnia in our findings, which could be proved in previous research. PDD patients slept shorter, had more night-time behavior disturbances, and were less satisfied with their sleep than patients with DLB [11], which could prove our finding. Restless legs syndrome (RLS), turn-over difficulty and pain [60] were in a higher proportion, which may be possible reasons for insomnia in PDD patients. The loss of hypocretinergic neurons in the hypothalamus could decrease hypocretin and disturb sleep-wake cycle regulation, resulted to insomnia in PDD [61]. What’s more, dopaminergic dysfunction with impairment of medial thalamic pain system in PD could cause RLS, and the associations between RLS and insomnia in patients with LBD remained to explore.

Patients with DLB demonstrated a poorer cognitive situation at their visit than patients with PDD. The ages at onset and at visit were both older in DLB than PDD, which was consistent with our multicenter findings [62] in Chinese memory clinics. Moreover, patients with DLB suffered more from hypertension and DM than PDD patients. Take as the fact that the older ages at onset and visit as well as the higher mean score of CDR in DLB into consideration, once again the possibility that there may be the demographic factors driving this cannot be discounted.

We provide the relatively large sample size and reliable information from outpatient records in current clinical research on DLB and PDD in early stage, while we recognize some limitations in this study. Firstly, the lack of specificity biomarkers (even pathological confirmation) and objective polysomnography could lead to bias in the determination of disease and RBD. And since all patients came from a single center, there existed a misclassification bias to some extents. Some relevant factors may be not taken into account in this research, which need further consideration in future.

## Conclusion

The presence and co-incidence of geriatric symptoms, including sleep disorders, autonomic nervous dysfunction, and NPS are common in patients with mild-moderate DLB or PDD. The APOE ε4 allele was represented in DLB, and is associated with hyperhidrosis and depression, but not global cognition, activitives of daily life, motor function and other neuropsychitric symptoms. While APOE ε4 allele plays no role in the clincal characteristics of PDD. These findings can signifiant improve the awareness of geriatric symptoms, and contribute to the healthcare management of mild-moderate DLB and PDD.

## Supplementary Information

**Additional file 1: Table S1.** Comparation of clinical core features between probable DLB and PDD. **Table S2.** Correlation between APOE ε4 allele, basic and clinical characteristics in probable DLB and PDD. **Table S3.** Associations between APOE ε4 allele and clinical core features in DLB and PDD.

## Data Availability

The data that support the findings of this study are available from the corresponding author upon reasonable request.

## Abbreviations

AD: Alzheimer’s disease
ADL: Activities of daily living
APOE: Apolipoprotein E
CDR: Clinical Dementia Rating
ChEIs: Cholinesterase inhibitors
CI: Cognitive impairment
95%CI: 95% confidential interval
DM: Diabetes mellitus
DLB: Dementia with Lewy bodies
EDS: Excessive daytime sleepiness
FLC: Fluctuating cognition
FoF: Fear of falling
H&Y stage: Hoehn-Yahr stage
HH: Hyperhidrosis
IQR: Interquartile range
LBD: Lewy body dementia
MDS-UPDRS III: Movement Disorder Society Unified Parkinson’s Disease Rating Scale part III
MMSE: Mini-Mental State Examination
MoCA: The Montreal Cognitive Assessment
NPI: Neuropsychiatric Inventory
OH: Orthostatic hypotension
ORs: Odds ratios
PCR: Polymerase chain reaction
PD: Parkinson’s disease
PDD: Parkinson’s disease dementia
RLS: Restless legs syndrome
RBD: Rapid eye movement sleep behavior disorder
SD: Standard deviation
VH: Visual hallucination
