# Supplementary material for: The presence and co-incidence of geriatric syndromes in older patients with mild-moderate Lewy body dementia
Source: BMC Neurol. 2022 Sep 19;22:355. doi: 10.1186/s12883-022-02897-7 (PMC9484208; doi:10.1186/s12883-022-02897-7)
Supplement: Supplementary file 1 — Additional file 1: Table S1. Comparation of clinical core features between probable DLB and PDD. Table S2. Correlation between APOE ε4 allele, basic and clinical characteristics in probable DLB and PDD. Table S3. Associations between APOE ε4 allele and clinical core features in DLB and PDD. [file 12883_2022_2897_MOESM1_ESM.docx]

**The presence and co-incidence of** **geriatric syndromes in older patients with mild-moderate Lewy body dementia**

Jinghuan Gan ^a#^, Zhichao Chen ^b#^, Shuai Liu ^c^, Zhihong Shi ^c^, Yiming Liu ^d^, Xiao-Dan Wang ^c^, Chunyan Liu ^e^, and Yong Ji ^a,c^ *

^a^ Department of Neurology, Beijing Tiantan Hospital, Capital Medical University, China National Clinical Research Center for Neurological Diseases, Beijing, China;

^b^ Department of Neurology, Beijing Friendship Hospital, Capital Medical University, Beijing, China;

^c^ Tianjin Key Laboratory of Cerebrovascular and of neurodegenerative diseases, Tianjin dementia institute, Department of Neurology, Tianjin Huanhu Hospital, Tianjin, China;

^d^ Department of Neurology, Qilu hospital, Shandong University, Jinan, China;

^e^ Department of Neurology, Aviation General Hospital, Beijing, China.

**Table S1** Comparation of clinical core features between probable DLB and PDD

|  | **DLB** | |  | **PDD** | |
| --- | --- | --- | --- | --- | --- |
|  | **APOE ε4**  **carriers (n=53)** | **APOE ε4**  **non-carriers (n=39)** |  | **APOE ε4**  **carriers (n=12)** | **APOE ε4**  **non-carriers (n=81)** |
| **FLC** | 15 (28.3%) | 11 (28.2%) |  | 2 (16.7%) | 7 (8.6%) |
| **VH** | 28 (52.8%) | 16 (41.0%) |  | 1 (8.3%) | 12 (14.8%) |
| **Parkinsonism** | 23 (43.4%) | 21 (53.8%) |  | 12 (100.0%) | 81 (100.0%) |
| **RBD** | 32 (60.4%) | 19 (48.7%) |  | 3 (25.0%) | 18 (22.2%) |

Abbreviations: DLB, dementia with Lewy bodies; PDD, Parkinson’s disease dementia; FLC, fluctuating cognition; VH, visual hallucination; RBD, rapid eye movement sleep behavior disorder.

**Table S2** Correlation between APOE ε4 allele, basic and clinical characteristics in probable DLB and PDD

|  | **DLB** | |  | **PDD** | |
| --- | --- | --- | --- | --- | --- |
|  | **Pearson Corr.** | **Partial Corr.** |  | **Pearson Corr.** | **Partial Corr.** |
| **Age at visit, years** | 0.097 | 0.044 |  | -0.011 | -0.059 |
| **Age at onset, years** | 0.091 | 0.048 |  | 0.016 | -0.044 |
| **MDS-UPDRS III** | -0.176 | -0.167 |  | 0.111 | 0.166 |
| **H&Y stage** | -0.057 | -0.046 |  | -0.071 | -0.067 |
| **ADL** | 0.198 | 0.180 |  | 0.167 | 0.128 |
| **MMSE** | -0.159 | -0.094 |  | -0.149 | -0.035 |
| Orientation | -0.103 | -0.028 |  | -0.065 | 0.060 |
| Registration | -0.071 | -0.009 |  | **-0.216*** | -0.201 |
| Attention and calculation | -0.128 | -0.094 |  | 0.025 | 0.059 |
| Recall | -0.024 | 0.036 |  | -0.045 | 0.016 |
| Language | -0.165 | -0.123 |  | -0.200 | -0.117 |
| Praxis | -0.116 | -0.071 |  | -0.158 | -0.103 |
| **MoCA** | -0.161 | -0.120 |  | -0.141 | -0.058 |
| Visuospatial/executive abilities | -0.040 | 0.026 |  | -0.185 | -0.139 |
| Naming | 0.062 | 0.100 |  | -0.009 | 0.023 |
| Attention | -0.172 | -0.130 |  | -0.069 | -0.030 |
| Language | **-0.270**** | **-0.264*** |  | -0.191 | -0.109 |
| Abstraction | -0.020 | -0.050 |  | -0.160 | -0.119 |
| Recall | -0.169 | -0.135 |  | 0.045 | 0.103 |
| Orientation | -0.085 | 0.031 |  | -0.053 | 0.038 |
| **NPI (total score)** | 0.038 | 0.031 |  | -0.070 | -0.036 |
| **Presence of core features** |  |  |  |  |  |
| FLC | 0.001 | -0.099 |  | 0.091 | 0.076 |
| VH | 0.117 | 0.081 |  | -0.063 | -0.058 |
| Parkinsonism | -0.103 | -0.128 |  | NA | NA |
| RBD | 0.116 | 0.080 |  | 0.022 | 0.037 |

The relationship between the presence of APOE ε4, basic and clinical characteristics of probable DLB and PDD were calculated by Pearson and Partial correlation analysis, the “R” was shown in this table. In the Partial correlation analysis, we adjusted the confounders of gender, education, habits of smoking and alcohol consumption, cardiometabolic conditions (heart disease, hypertension, diabetes mellitus, stroke) and CDR. **p* < 0.05, ***p* < 0.01.

Abbreviations: DLB, dementia with Lewy bodies; PDD, Parkinson’s disease dementia; APOE, Apolipoprotein E; MDS-UPDRS III, Movement Disorder Society Unified Parkinson’s Disease Rating Scale part III; H&Y stage, Hoehn-Yahr stage; ADL, activities of daily living; MMSE, Mini-Mental State Examination; MoCA, the Montreal Cognitive Assessment; NPI, Neuropsychiatric Inventory; FLC, fluctuating cognition; VH, visual hallucination; RBD, rapid eye movement sleep behavior disorder; CDR, Clinical Dementia Rating.

**Table S3** Associations between APOE ε4 allele and clinical core features in DLB and PDD

| **Clinical core**  **features** | **DLB**  **(APOE ε4 carriers vs. non-carriers)** | | |  | **PDD**  **(APOE ε4 carriers vs. non-carriers)** | | |
| --- | --- | --- | --- | --- | --- | --- | --- |
|  | **OR** | **95%CI** | **P-value** |  | **OR** | **95%CI** | **P-value** |
| **FLC** | 0.690 | 0.229-2.079 | 0.510 |  | 3.016 | 0.399-22.788 | 0.285 |
| **VH** | 1.409 | 0.560-3.550 | 0.467 |  | 0.558 | 0.055-5.609 | 0.620 |
| **Parkinsonism** | 0.540 | 0.214-1.373 | 0.197 |  | NA | NA | NA |
| **RBD** | 1.413 | 0.574-3.479 | 0.451 |  | 1.284 | 0.284-5.809 | 0.745 |

The models were adjusted gender, age at visit, education, habits of smoking and alcohol consumption, heart disease, hypertension, diabetes mellitus, stroke and CDR.

Abbreviations: DLB, dementia with Lewy bodies; PDD, Parkinson’s disease dementia; ORs, odds ratios; 95%CI, 95% confidence interval; APOE, Apolipoprotein E; FLC, fluctuating cognition; VH, visual hallucination; RBD, rapid eye movement sleep behavior disorder; CDR, Clinical Dementia Rating.
